# WMCA-Net: Wavelet Multi-Scale Contextual Attention Network for Segmentation of the Intercondylar Notch

**DOI:** 10.3390/bioengineering13020236

**Published:** 2026-02-18

**Authors:** Yi Wu, Xiangxin Wang, Hu Liu, Quan Zhou, Lingyan Zhang, Yujia Zhou, Qianjin Feng

**Affiliations:** 1 School of Biomedical Engineering, Southern Medical University, Guangzhou 510515, China; yiwu410@126.com (Y.W.); liuhu_smu@outlook.com (H.L.); 2 School of Computer Science and Engineering, Southeast University, Nanjing 210096, China; 230208696@seu.edu.cn; 3 Department of Medical Imaging, The Third Affiliated Hospital, Southern Medical University, Guangzhou 510630, China; zhouquan3777@smu.edu.cn (Q.Z.); 18819818005@163.com (L.Z.)

**Keywords:** intercondylar notch segmentation, MRI segmentation, knee joint segmentation, wavelet decomposition, multi-scale feature learning, attention mechanism

## Abstract

Accurate segmentation of the intercondylar notch of the femur is of great significance for the diagnosis of knee joint diseases, surgical planning, and anterior cruciate ligament (ACL) reconstruction. Among them, the obvious anatomical heterogeneity, the interference of structurally similar tissues, and the blurred boundaries in MRI images make the segmentation of the intercondylar notch challenging. The segmentation of the intercondylar notch is often regarded as a standard semantic segmentation problem, but doing so leaves the inherent high-order internal variation and low-contrast features of its anatomical structure unresolved. We proposed a new Wavelet Multi-scale Contextual Attention Network (WMCA-Net). We have coordinated the Shallow High-frequency Feature Dense Extraction Block (SHFDEB) and Wavelet Split and Fusion Block (WSFB) modules with each other. The SHFDEB intensively extracts high-frequency detailed features at the shallowest layer of the network, while the WSFB effectively splits and fuses features at various resolutions, suppressing noise while better preserving the high-frequency detailed structural information we need. The Multi-scale Depth-wise Convolution Block (MDCB) captures cross-scale features from the narrow intercondylar notch (5–8 mm wide) to the surrounding femoral structure (approximately 50 mm diameter), dynamically adapting to different morphologies, including pathological changes caused by osteophyte formation. The Contextual-Weighted Attention Module (CWAM) establishes long-term semantic associations between fuzzy regions and clear anatomical landmarks by precisely locating uncertain regions through foreground and background decomposition. The Dice Similarity Coefficient of WMCA-Net on the intercondylar notch dataset is 93.16%, and the 95% Hausdorff Distance is 1.42 mm, demonstrating its advanced segmentation performance and good anatomical adaptability.

## 1. Introduction

The accurate segmentation of the intercondylar notch of the femur, also known as the intercondylar fossa, plays a crucial role in the diagnosis and treatment of knee joint diseases, as well as in surgical plans such as anterior cruciate ligament (ACL) reconstruction and arthroscopic surgery [1,2,3]. Notch segmentation based on 2D MR images can provide quantification for preoperative key morphological quantitative measurements, such as slice width, volume, slice width index and other morphological parameters, and can also provide objective data support for ACL injury risk assessment [4,5,6,7]. This technology can also provide positioning for the implanted graft, avoiding the collision between the implant and the bone caused by overly narrow incisions, thereby reducing the risk of postoperative complications such as joint fibrosis or graft retearing in advance. Manual segmentation of the intercondylar notch is time-consuming and labor-intensive, and shows a large inter-observer variability of 15–20%, reducing the reliability of the data. In contrast, the automatic segmentation technology improves efficiency, enhances reproducibility, and provides more comprehensive and robust data for the evaluation of joint stability [8,9].

**Figure 1:**
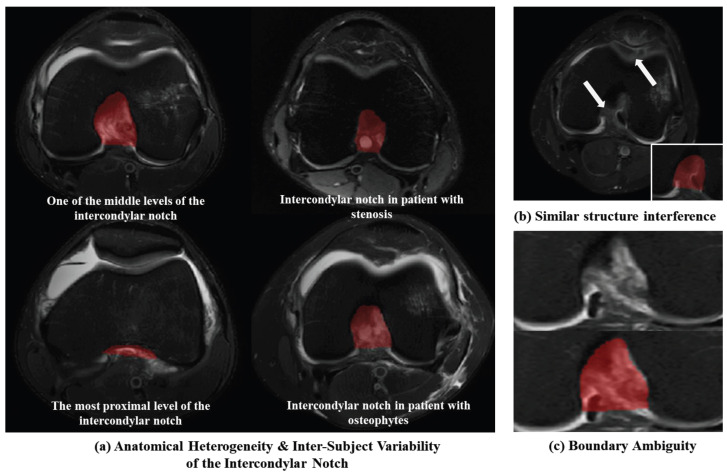
The major challenges of accurate automatic segmentation of the intercondylar notch.The red area represents the intercondylar notch region. (**a**) Anatomical Heterogeneity and Intra-Subject Variability of the Intercondylar Notch: There are marked variations between the proximal and middle regions of the intercondylar notch within the same individual, and significant differences are observed between patients with intercondylar notch stenosis and those with osteophytes. (**b**) Interference from Structurally Similar Tissues: Adjacent anatomical structures share comparable imaging appearances and geometrical shapes with the intercondylar notch. (**c**) Boundary Ambiguity: The margins of the intercondylar notch are in close proximity to the cruciate ligaments and synovial tissues, exhibiting low contrast.

In recent years, convolutional neural network (CNN)-based deep learning has become widely adopted for various segmentation tasks, owing to its state-of-the-art representational performance. In spite of that, accurate and robust automatic segmentation of the intercondylar notch remains challenging due to several key factors (Figure 1):

(A) Anatomical Heterogeneity and Intra-Subject Variability of the Intercondylar Notch: The intercondylar notch is an irregular concave structure at the distal end of the femur, which shows rich diversity in morphology. In the same subject, the cross-sectional area of the proximal and middle parts of the intercondylar notch varies greatly, up to 30%. Among different individuals, there are also significant differences in the width (the normal range is approximately 12–18 mm, and in narrow cases, it may be less than 10 mm), depth and contour shape of the intercondylar fossa. In addition, approximately 30% of people will develop osteophytes due to osteoarthritis or post-traumatic bone remodeling, which leads to pathological changes in the morphology of the incisions. This exacerbates the anatomical complexity of the incisions on MR Images and poses a significant challenge to medical image segmentation tasks.;
(B) Interference from Structurally Similar Tissues: The adjacent anatomical structures and intercondylar notches are very similar in imaging color and shape, which is very likely to lead to classification errors. The scar tissue after anterior cruciate ligament injury may fill the intercondylar fossa, resulting in a difference of less than 10% in MRI signals between normal tissue and pathological tissue, causing an overlap of gray values and making segmentation more difficult;
(C) Boundary Ambiguity: The edges of the intercondylar notch closely adjoin the cruciate ligaments and synovial tissues. In sagittal MRI, the bone–ligament interface exhibits blurred boundaries due to mixed signal intensities. This low contrast characteristic makes it challenging to acquire sufficient high-quality annotated data for model training.

In response to the existing challenges, this paper specifically designs the network architecture WMCA-Net for the effective and robust segmentation of the intercondylar notch.

### 1.1. Related Work

#### 1.1.1. Medical Image Segmentation

Deep learning technology has developed significantly in the field of medical image segmentation, and its performance is obviously superior to traditional methods. Ronneberger et al. proposed the U-Net [10] architecture, whose encoder–decoder structure and skip connections integrate multi-scale features, making the U-Net architecture a benchmark model for medical image segmentation. However, standard downsampling may lose details that are crucial for blurred boundaries, and fixed convolution kernels cannot adapt to regions with extreme scale variations such as the intercondylar notch. He et al. proposed that ResNet [11] solves the vanishing gradient problem in deep networks through residual connections. Huang et al. proposed a dense structure [12] that achieves feature reuse through a dense connection mechanism. Zhang et al. integrated ResNet with U-Net structures into ResUNet [13], and Li et al. [9] applied it in the segmentation of the intercondylar fossa and achieved very good results. Zhou et al. improved the original U-Net in unet++ [14] through nested skip joins and dense supervision mechanisms, enabling the model to adaptively select the optimal feature fusion path between the codec layers. Chen et al. first proposed the atrous spatial pyramid pooling (ASPP) in DeepLabv3+ [15], capturing multi-scale context information by controlling the receptive field through expansion rate, which is particularly suitable for medical images with different target scales. Szegedy et al. designed InceptionNet [16] to achieve multi-scale feature extraction within a single module, employing parallel multi-scale convolution kernels and pooling operations. This not only reduces parameter counting but also enhances feature diversity, enabling powerful modeling of complex textures in medical images. Isensee et al. developed nnUNet [17] to achieve robust performance across multimodal and multi-scale medical data by automating a series of processes including data preprocessing, network architecture search, and training strategy optimization. In various subdivided medical image challenges, nnUNet outperforms most dedicated models.

After Dosovitskiy et al. proposed the Vision Transformer (ViT) [18] for general computer vision, Chen et al. applied it to explore the application of medical image segmentation to address the urgent need for long-distance feature extraction in high-resolution heterogeneous medical data. However, the high computational complexity of the self-attention mechanism also poses challenges to the dense pixel-level prediction in medical images. Chen et al. (2021) integrated convolutional neural networks with transformers to form a hybrid framework called TransUNet [19]. This way, not only can the local anatomical detail extraction ability of the CNN be combined with the global structural relationship reasoning of the transformer, but their respective advantages can also be brought into play. TransUNet achieved excellent multi-organ segmentation performance on the Synapse CT dataset. Due to the complete Transformer decoder, the computational overhead is very high. Cao et al. [20] developed Swin-UNet with a hierarchical Swin-Transformer backbone and shift window mechanism to reduce self-attention complexity from $O(n^{2})$ to O(n), demonstrated the efficient processing of 2D/3D medical data, which can be used for the segmentation of skin lesions and brain tumors.Although the symmetrical U-Net structure in Swin UNet can capture the global context while maintaining the retention of spatial details, due to the limited inductive bias, careful tuning for small datasets is required. Although the symmetrical U-Net structure in Swin UNet can capture the global context while maintaining the retention of spatial details, due to the limited inductive bias, careful tuning for small datasets is required. Hatamizadeh et al. proposed UNETR [21], which is specifically designed for 3D volume segmentation. It targets abdominal organs in CT scans, uses a transformer encoder to simulate the spatial relationship of the entire convolution, and employs a CNN decoder for pixel-level refinement. Because UNETR processes anisotropic 3D data and does not use patch-based approximations, the model has a huge demand for GPU memory. Hatamizadeh et al. suggest pre-training large-scale unlabeled medical datasets to alleviate annotation scarcity.

However, the above-mentioned general medical segmentation algorithms have some inherent limitations in structural design when considering segmental intercondylar notches. Models based on the CNN mainly rely on fixed small-sized convolution kernels (such as 3 × 3), making it difficult to establish global dependencies between distant pixels. Transformer-based models effectively capture global context information through self-attention mechanisms, but the computational cost is very high. Moreover, most of the above-mentioned segmentation methods adopt downsampling to expand the receptive field and extract high-order semantic features, resulting in the loss of high-frequency detail information. The intercondylar fossa itself is very small, usually only 5–8 mm wide, and its boundary often appears as an uncertain area with gradually changing gray levels on MRI images. The absence of such details directly leads to rough, discontinuous and even boundary-displaced edges in the segmented intercondylar notches. Although models such as U-Net fuse shallow details and deep semantic information by skipping connections, how to effectively fuse features at different levels remains a challenge.

#### 1.1.2. The Intercondylar Notch Segmentation

Accurate intercondylar fossa segmentation is an important foundation for the diagnosis of knee joint diseases and the planning of anterior cruciate ligament reconstruction surgery. Rollet et al. [22] and Vaswani et al. [23] developed an intuitive and easy-to-implement method for measuring intercondylar notches. These traditional methods have strong operator dependence and poor repeatability, and it is difficult to obtain complete 3D structural information. With the development of technology, deep learning methods have significantly improved the segmentation performance.

Li et al. [9] used the Res-UNet model to segment the intercondylar fossa and obtained high-precision automatic segmentation results, which improved the clinical efficacy. However, this method not only requires a large amount of labeled data, but also has limited adaptability to cases with significant differences in anatomical structures, making it difficult to capture the subtle morphological differences in the grooves of patients with osteophytes. Ghidotti et al. [24] combined deep learning with statistical shape modeling (SSM), which improved segmentation accuracy and enhanced anatomical interpretability. Enhancing the robustness of morphological variations by capturing the main change patterns of anatomical structures can better handle individual differences, but the implementation form of this algorithm is more complex.

The difficulty of knee joint segmentation, especially cartilage segmentation, lies in the relatively low contrast between cartilage and surrounding tissues in MRI [25]. Norman et al. [26] implemented a segmentation method for knee cartilage and meniscus based on U-Net, and the DSC values of the model ranged from 0.77 to 0.88. Jonmohamadi et al. [27] also achieved high segmentation accuracy by applying U-NET ++ to the multi-structure segmentation of arthroscopic images. It has been demonstrated that the U-shaped structure is adaptable to complex structures, but it still shows deviations in challenging pathological cases.

Later, Ambellan et al. [28] proposed an integrated method that combines statistical shape knowledge with a 2D/3D CNN for knee osteocartilage segmentation, using anatomical priors to enhance the model’s robustness to abnormal structures. The strong complexity of the algorithm also invisibly increased the computational cost. Ebrahimkhani et al. [29] proposed a hybrid learning framework that combines differential isomorphic mapping with deep elements and manual elements, effectively fusing the expressive power of deep features and the interpretability of traditional features. Ebrahimkhani et al. demonstrated the stability of the model in the face of cross-center and multi-time datasets, but this also brought about complex feature engineering and model fusion processes, requiring the redesign of parameters for different datasets.

The aim of enhancing the attention mechanism of the boundary [30,31,32,33] is to increase its sensitivity to key areas by weighting in the channel or spatial dimensions. However, its main limitation lies in the lack of explicit utilization and decomposition of the image frequency domain information, making it difficult to distinguish between noisy high-frequency signals and the true anatomical boundaries. Meanwhile, wavelet transform, with its multi-resolution analysis capability, has already been widely applied in medical image denoising and enhancement, and as a feature extractor, and has recently begun to be embedded as a preprocessing or feature enhancement module in deep learning segmentation frameworks [34,35]. However, it has not been deeply and end-to-end co-designed with the dynamic multi-scale context modeling mechanism and the attention mechanism for semantic ambiguous regions. We propose WMCA-Net, which designs a multi-dimensional collaborative modular architecture that integrates wavelet transform with a multi-scale semantic extraction and attention mechanism, for noise and enhancement of spatial domain and frequency domain details features, while maintaining boundary accuracy and scale adaptability.

### 1.2. Overall Network Architecture Overview

**Figure 2:**
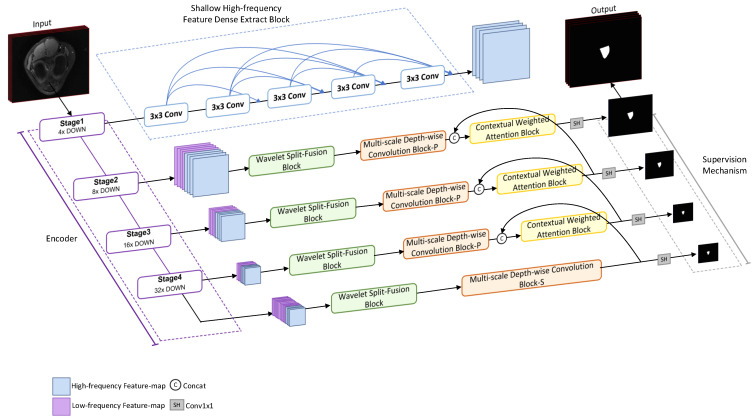
Overall architecture of the proposed intercondylar notch segmentation method.

In this study, we propose a novel wavelet multi-scale context attention network, WMCA-Net, to achieve automatic segmentation of the intercondylar fossa. In Figure 2, we can see that this architecture is based on the classic U-shaped framework and has several key components: the Shallow High-Frequency Feature Dense Extraction Block (SHFDEB), the Wavelet Split and Fusion Block (WSFB), the Multi-scale Depth-wise Convolution Block (MDCB), and the Contextual-Weighted Attention Block (CWAB). In the encoder, ResNet50 or Transformer can be selected as the backbone network to extract features of different resolutions from the input image. The SHFDEB module acquires high-frequency information rich in details from the shallowest layer. During the decoding process, the features from Stage2 to Stage4 are first concatenated with the downsampled high-frequency detail features, and then effective multi-frequency fusion is carried out through the WSFB. The fused features then capture multi-scale context information through the MDCB and are ultimately processed by the CWAB, with the uncertain regions weighted through a deep supervision mechanism.

The synergy of these modules enables WMCA-Net to effectively address the specific challenges in the segmentation of intercondylar notches of the knee joint: SHFDEB and WSFB alleviated boundary blurring (Challenge C), MDCB addressed anatomical heterogeneity and inter-individual differences (Challenge A), and CWAB reduced the interference and misclassification of structurally similar tissues (Challenge B). By making full use of multi-scale and frequency-aware features, the segmentation performance of the network for intercondylar notches and various types of knee joint MRI images has been improved.

### 1.3. Contributions

The main contributions of this paper are as follows:

We propose WMCA-Net, a novel end-to-end wavelet multi-scale contextual attention network that achieves state-of-the-art intercondylar notch segmentation and demonstrates strong generalization across diverse datasets.

- A Multi-scale Depth-wise Convolution Block (MDCB) that synchronously captures cross-scale features, effectively addressing the extreme anatomical heterogeneity and pathological variations (Challenge A) within the intercondylar notch;
- A Context-Weighted Attention Module (CWAM) that accurately locates and enhances uncertain boundary regions, establishing long-range semantic associations to mitigate misclassification due to similar tissues and overlapping grayscale values (Challenge B);
- The Shallow High-Frequency Feature Dense Extraction Block (SHFDEB) and Wavelet Split and Fusion Block (WSFB) to preserve crucial high-frequency details and improve the recognition of blurred boundaries, thereby solving the problem of boundary ambiguity (Challenge C).

## 2. WMCA-Net

As shown in Figure 2, we propose the lightweight network WMCA-Net for the intercondylar fossa segmentation problem. WMCA-Net is based on the standard Transformer backbone network and consists of four core components: the SHFDEB module, the WSFB module, the MDCB module and the CWAB module. This model aims to supplement the problem of lost details when the network deepens with high-frequency detail features. Specifically, the SHFDEB module extracts the detailed features of an image from the shallow layer of the encoder through a densely connected convolutional structure. Then, the WSFB module adopts the Haar wavelet transform to further fuse the high-frequency features with the low-frequency features rich in semantics, enhancing the semantic understanding ability while retaining the boundary details. Then, the MDCB module extends the receptive field through multi-scale large kernel depth-separable convolution, effectively capturing the cross-scale association between the intercondylar fossa and the surrounding structures. Finally, the CWAB module dynamically weights the uncertain regions under the guidance of semantic features, reducing classification errors caused by blurred boundaries.

### 2.1. Shallow High-Frequency Feature Dense Extraction Block (SHFDEB)

To avoid the loss of high-frequency details during downsampling, we introduce SHFDEB at the shallowest encoder stage (the first stage). It adopts a dense connection of separable convolution structures to capture and retain these fine details from the original input, preserving the high-frequency detail information in the spatial domain for the subsequent downsampling stages.

### 2.2. Wavelet Split and Fusion Block (WSFB)

**Figure 3:**
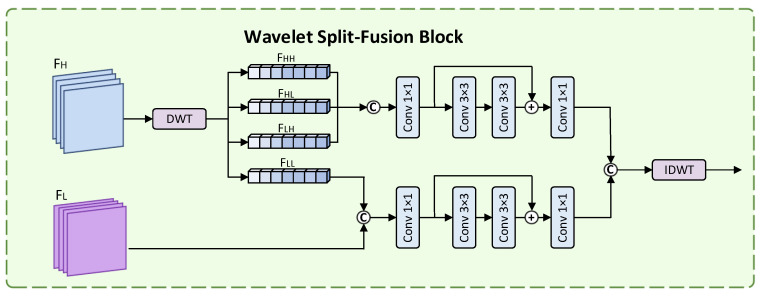
Wavelet Split-Fusion Block.

To counteract the boundary blurring caused by progressive downsampling, we embed an WSFB after each encoder stage. It uses wavelet transformation for frequency domain analysis and feature recombination. We chose the Haar wavelet because of its high computational efficiency, and its ability to capture the clear boundary features of obvious anatomical structures in the image, and can decompose features more directly and efficiently, without introducing excessive computational complexity. It simultaneously achieves an effective combination of high-frequency details and low-frequency semantic information, which not only enhances the detail expression of the intercondylar fossa edge but also improves the feature discrimination ability of the boundary blur area. As shown in Figure 3, the core steps of this module are as follows:

(1) Wavelet Decomposition:
  For T2-weighted knee intercondylar notch MRI images, the single-layer Haar wavelet transform can be applied to decompose and generate one low-frequency approximate coefficient and three high-frequency detail coefficients (LH, HL, and HH), and the resolution of each coefficient is half of the original image. Among them, the low-frequency approximation coefficient LL encodes global semantic information, such as the overall morphology of the distal femur and tibia, providing a macroscopic anatomical background for the localization of the intercondylar fossa. LH, representing the horizontal high-frequency coefficient, can capture the intensity changes in the horizontal direction and highlight the left and right boundaries of the intercondylar fossa relative to the medial and lateral condyles of the femur. HL, also known as the vertical high-frequency coefficient, can detect the details of the vertical edge and is helpful for the model to identify the upper and lower boundaries of the intercondylar fossa. HH, diagonal high-frequency coefficient, shows the diagonal texture recognition pattern of meniscus fibers or ACL attachment points, enhancing the ability to distinguish fine features.
(2) Dual-path Differentiated Processing:
  - High-frequency Detail Processing Pathway:
    The high-frequency coefficients (LH, HL, and HH) contain the key boundary details of intercondylar notch segmentation, but are prone to carrying MR Image noise artifacts caused by motion. To address this issue, we apply a soft threshold function (threshold = 0.05) to each coefficient to suppress noise, achieving a balance between random noise removal and detail retention. Then we concatenate the three-channel feature maps of the high-frequency signals with reduced noise of LH, HL and HH coefficients, and connect them with BN and ReLU through a lightweight convolution unit, namely two 3 × 3 convolution units, to extract the deep correlation features. Among them, $F_{H}$ is compressed to 1/4 to ensure size compatibility with the fused low-frequency feature $F_{L}$.
  - Low-Frequency Semantic Processing Pathway:
    The low-frequency approximation coefficient (LL) contains global structural semantics but lacks the richness of detailed expression. To enhance this, the shallow low-frequency feature fusion of LL and CNN encoders can not only make good use of the multi-scale semantics extracted by baseline to strengthen the context information but also enrich the basic structure information necessary for the precise anatomical segmentation model.
(3) High-Low Frequency Feature Fusion:
  In the case of resolution mismatch, in order to effectively integrate high-frequency detail feature maps and low-frequency semantic feature maps, we adopted the following processing method. First, downsample the high-frequency feature map to match its resolution with that of the low-frequency feature map, and align its spatial dimensions. Then connect the two mappings along the channel dimension. Finally, a 1 × 1 convolution is performed on the output after splicing to compress the features of each channel. The batch normalization (BN) layer and the ReLU activation function are used for feature normalization and nonlinear enhancement to obtain the final fused feature output.

### 2.3. Multi-Scale Depth-Wise Convolution Block (MDCB)

To handle the extreme scale variation between the small intercondylar notch and surrounding femoral structures, we design multi-scale deep convolution blocks -Parallel and -Serial, using multi-scale branches to handle size differences and large convolution kernel bottleneck modules to adapt to morphological diversity. The MDCB module can effectively balance the feature extraction of large-scale anatomical structures (such as the femoral condyle) and small-scale targets (such as intercondylar incisions), improve the adaptability to diverse intercondylar incisions (including type a, type u and type w), and thereby enhance the segmentation accuracy of small-volume and pathological intercondylar notchs.

**Figure 4:**
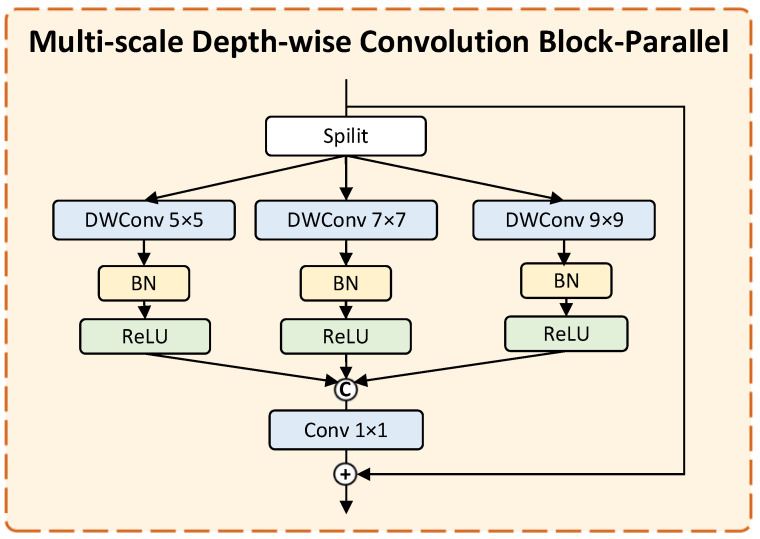
Multi-scale Depth-wise Convolution Block-Parallel Module.

**Figure 5:**
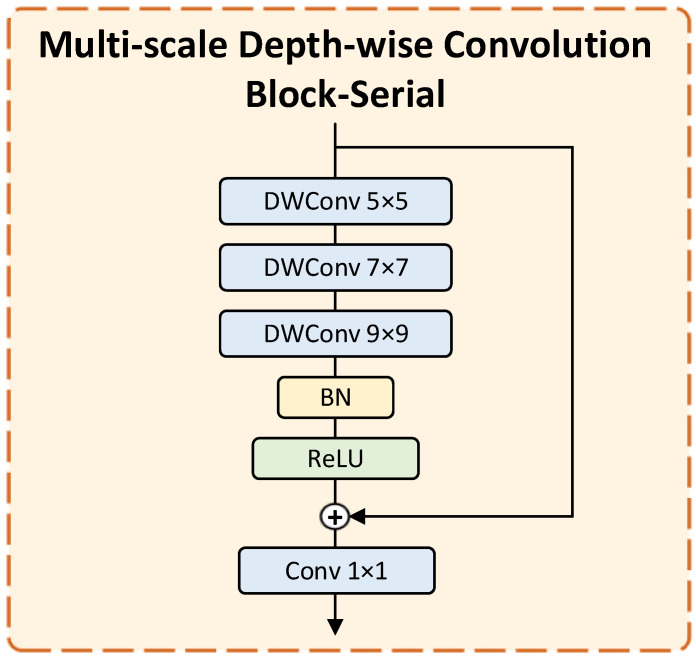
Multi-scale Depth-wise Convolution Block-Serial Module.

(1) Multi-scale Depth-wise Convolution Block-Parallel (MDCB-P):
  As shown in Figure 4, the MDCB-Parallel (MDCB-P) employs parallel branches with depth-wise convolutions of different kernel sizes to capture multi-scale contextual features simultaneously, enhancing the model’s adaptability to structures of varying scales.
(2) Multi-scale Depth-wise Convolution Block-Serial (MDCB-S):
  As shown in Figure 5, the MDCB-Serial (MDCB-S) acts as a bottleneck module, sequentially applying large-kernel depth-wise convolutions to further expand the receptive field and model long-range dependencies in deep feature maps.

### 2.4. Context-Weighted Attention (CWAM)

**Figure 6:**
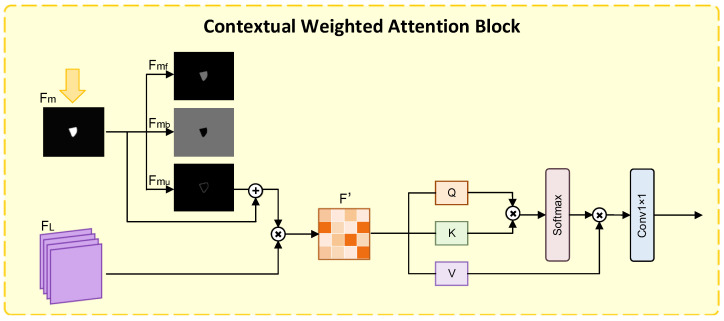
Contextual Weighted Attention Block.

We designed a context-weighted attention (CWAM) module to quantify the uncertain regions based on saliency maps, enhancing the semantic and contextual interaction between the uncertain regions and the foreground/background, thereby improving the segmentation accuracy of the uncertain regions. The structure of this module is shown in Figure 6.

(1) Uncertain Region Definition and Enhancement:
  First, $F_{m}$ is upsampled to align its size with the feature $F_{L}$. Then, an adaptive threshold (e.g., 0.5) is used to decompose the upsampled $F_{m}$ feature into three distinct regions: foreground map $m_{f}=\max\left(F_{m}-0.5,0\right)$ (saliency value >0.5, corresponding to high-confidence intercondylar fossa regions), background map $m_{b}=\max\left(0.5-F_{m},0\right)$ (saliency value <0.5, corresponding to high-confidence background regions), and uncertain region map $m_{u}=0.5-\left|F_{m}-0.5\right|$ (saliency value close to 0.5, corresponding to grayscale gradient regions). By enhancing the distinction between foreground and background, the extent of the uncertain region can be precisely defined.
  Furthermore, the uncertain region mask $m_{u}$ is added element-wise to the upsampled deep features $F_{m}$. In this way, high-level semantic supervision signals can be injected into the uncertain region, effectively reducing misclassifications caused by feature blurring.
(2) Contextual Weighted Feature Fusion:
  The enhanced uncertain region features are concatenated with the feature $F_{L}$ along the channel dimension to obtain the fused feature $F^{\prime }$. $F^{\prime }$ is fed into three parallel 1×1 convolutional layers (i.e., *Q*, *K*, and *V* projection layers) to generate query vector *Q*, key vector *K*, and value vector *V* respectively. Here, *Q* captures the feature requirements of the current pixel, *K* encodes feature information of all pixels, and *V* stores the feature values to be weighted. Finally, the scaled dot-product attention mechanism is used to calculate self-attention weights. The module effectively models long-distance semantic associations between uncertain regions and foreground/background—for example, through self-attention weights, feature information of the intercondylar fossa foreground is transmitted to adjacent uncertain regions, assisting in category determination of uncertain regions.

### 2.5. Loss Function

The intercondylar notch, as a smaller structure within the knee joint, accounts for only approximately 5% to 8% of the total pixel area. We use a special hybrid loss function for the intercondylar notch. Through the weighted fusion of Dice Loss and Focal Loss, a dual improvement in the segmentation accuracy of small structures and boundary accuracy is achieved. The overall formula is

(1) Loss=α·DiceLoss+β·FocalLoss

This loss function can not only leverage the optimization advantage of Dice Loss on regional overlap degree, but also focus on difficult-to-distinguish samples through Focal Loss, maximizing the synergistic effect of the two types of losses. The design logic and functions of each loss term are as follows:

(2) $$\mathrm{Dice}\;\mathrm{Loss}=1-\frac{2\cdot |Y\cap \hat{Y}|}{\left|Y|+\right|\hat{Y}|}$$

(3) $$\mathrm{Focal}\;\mathrm{Loss}=-\frac{1}{N}\sum_{k=1}^{K}\sum_{i=1}^{N}\left[\alpha_{k}{\left(1-P_{ik}\right)}^{\gamma }\log\left(P_{ik}+\varepsilon \right)\right]$$

In Equation (3), *N* represents the total number of pixels in the image, and *K* represents the number of segmentation categories (in the intercondylar notch segmentation, it is divided into two categories: background and intercondylar notch; in the knee joint segmentation, it is divided into five categories: background, femur, tibia, meniscus, and cruciate ligament), ensuring coverage of the key structures of the knee joint. $P_{ik}$ is the predicted probability that the *i*-th pixel belongs to the *k*-th category, directly reflecting the model’s confidence in determining the pixel category. $\alpha_{k}$ is the balanced weight of the *k*-th category, calculated based on the proportion of category pixels. When the intercondylar fossa is regarded as a minor category, its $\alpha_{k}$ value is significantly higher than that of the background, which can further enhance the loss contribution of the minor category samples.

γ is the focusing parameter. When the pixel is an easily classifiable sample (such as the $P_{ik}$ of the background pixel approaching 1), ${\left(1-P_{ik}\right)}^{\gamma }$ approaches 0, and the loss contribution is significantly suppressed. When pixels are difficult-to-classify samples (such as the $P_{ik}$ of the edge pixels of the intercondylar notch being close to 0.5), ${\left(1-P_{ik}\right)}^{\gamma }$ approaches 1, and the loss contribution is completely retained, thereby achieving the goal of focusing on learning difficult samples. $\varepsilon =1\times 10^{-5}$ is the smoothing term, which is used to avoid the calculation error caused by log(0) when $P_{ik}=0$ and ensure the numerical stability of the loss function.

The hybrid loss function can, through the synergistic effect of Focal Loss and Dice Loss, not only solve the problem of multi-class imbalance but also ensure the integrity and boundary clarity of the intercondylar fossa region, providing a precise optimization direction for model training.

## 3. Experiment Configurations

### 3.1. Dataset

This study adopted a dataset of 630 knee joint MRI cases collected by the PACS system of the Third Affiliated Hospital of Southern Medical University, including 398 patients with anterior cruciate ligament (ACL) injury and 232 control cases without ACL injury. The data covered two clinical routine sequences: ① Axial proton density-weighted—fat suppression rapid spin echo sequence (PDW-SPIR), where the collected parameters were field of view 160 mm × 160 mm × 92 mm, echo time 30 ms, repeat time 3000 ms, layer thickness 4 mm, and flip angle 90°, a total of 391 cases; ② Axial T2-weighted fat-suppressed fast spin echo sequence (T2W-SPIR), where the collected parameters were a field of view of 160 mm × 160 mm × 105 mm, echo time of 65 ms, repeat time of 2768 ms, layer thickness of 4 mm, and a rotation angle of 90°, totaling 239 cases. All images were precisely manually annotated based on anatomical landmarks by radiologists with more than 5 years of experience in knee joint imaging diagnosis using ITK-SNAP software. In addition, this study also adopted the public dataset of SKI-10 knee joint segmentation [36] as the external validation set. This dataset contains 3D double-echo steady-state precession sequence (3D DESS) images of 10 healthy volunteers with a spatial resolution of 0.7 mm layer thickness and a matrix of 384 × 384, and each case contains 120–150 layers of coronal plane slices. The annotation categories cover the femur, tibia, meniscus, cruciate ligament and background, providing a standardized evaluation benchmark for multi-tissue segmentation of the knee joint.

### 3.2. Implementation Details

The training set was augmented by random rotation ($\pm 15^{\circ }$), horizontal flipping (0.5 probability), and elastic deformation (α=800, σ=20). The optimizer was AdamW with weight decay $=1\times 10^{-4}$, and the initial learning rate was set to $1\times 10^{-4}$ with cosine annealing decay (period =1000 epochs, minimum learning rate $=1\times 10^{-6}$). An early stopping mechanism was employed: Training was halted and the optimal weights were loaded if the Dice Similarity Coefficient (DSC) on the validation set did not increase for 20 consecutive epochs.

### 3.3. Evaluation Metrics

We evaluated the performance of this method using three metrics for intercondylar notch segmentation, DSC (%), IoU (%), and HD95 (mm); and two metrics for knee joint segmentation, DSC (%) and ASSD (mm).

- Dice Similarity Coefficient (DSC): The DSC is used to evaluate the similarity of the foreground regions in two images according to Equation (4):
  (4) $$\mathrm{DSC}=\frac{2\cdot TP}{2\cdot TP+FN+FP}$$
  where TP (True Positive) represents the number of target pixels correctly classified, TN (True Negative) represents the number of background pixels correctly classified, FP (False Positive) represents the number of background pixels mistakenly classified as targets, and FN (False Negative) is the number of target pixels mistakenly classified as background.
- Intersection over Union (IoU): Also known as the Jaccard Index, the IoU measures the degree of overlap between predicted segmentation and ground truth by calculating the ratio of intersection to union according to Equation (5):
  (5) $$\mathrm{IoU}=\frac{TP}{TP+FN+FP}$$
- 95% Hausdorff Distance (HD95): The 95% Hausdorff distance excludes the top 5% of maximum distance values, providing a more robust and representative assessment of overall boundary accuracy according to Equation (6):
  (6) $$\mathrm{HD}95\left(B_{Y},B_{\hat{Y}}\right)=\max{\left(\underset{p\in B_{Y}}{\sup}\underset{q\in B_{\hat{Y}}}{\inf}d\left(p,q\right),\underset{q\in B_{\hat{Y}}}{\sup}\underset{p\in B_{Y}}{\inf}d\left(p,q\right)\right)}_{95\%}$$
  where $B_{Y}$ and $B_{\hat{Y}}$ represent the boundary point sets of the ground truth and predicted segmentation, respectively, and d(p,q) denotes the Euclidean distance between points *p* and *q*.
- Average Symmetric Surface Distance (ASSD): The ASSD calculates the average distance of all point pairs in two boundary sets, measured in millimeters (mm) according to Equation (7):
  (7) $$\mathrm{ASSD}=\frac{1}{|B_{Y}\left|+\right|B_{\hat{Y}}|}\left(\sum_{p\in B_{\hat{Y}}}\min_{q\in B_{Y}}d\left(p,q\right)+\sum_{q\in B_{Y}}\min_{p\in B_{\hat{Y}}}d\left(p,q\right)\right)$$
  where $|B_{Y}|$ and $|B_{\hat{Y}}|$ denote the numbers of boundary points in the ground truth and predicted segmentation, respectively.

### 3.4. Comparative Method

To demonstrate the effectiveness and superiority of the proposed WMCA-Net framework for automatic intercondylar notch segmentation, we conducted comprehensive experiments and comparisons with SOTA methods. We first evaluated our method by comparing it with a series of classic U-Net encoder-decoder variants, including UNet [10], UNet++ [14], and Attention UNet [30]. Then we compared with several attention-enhancing networks, such as SANet [32], PraNet [31], and UACANet [33], which are adept at handling boundary blurring and multi-scale anatomy by using channel, spatial and recurrent attention mechanisms. We also compared with Transformer-based architectures, including ransUNet [19] and Swin UNet [20], as well as the adaptive framework nnUNet [17], which is renowned for its leading performance in various medical image analysis tasks. The experimental results show that the method we proposed has achieved the most advanced performance.

## 4. Results and Analysis

We use two independent datasets to conduct a comprehensive performance evaluation of the proposed WMCA-Net. In-house datasets are used to verify the overall effectiveness of this method and evaluate the impact of each core module on the entire model. The performance of the model on the public dataset demonstrates the generalization ability of WMCA-Net across different data sources. All the evaluation indicators in this paper are the average values of five-fold cross-validation.

### 4.1. Results on In-House Dataset

We evaluated the effectiveness of WMCA-Net in two aspects. Firstly, a transverse comparative analysis was conducted on the results of quantitative and qualitative intercondylar notch segmentation using the existing state-of-the-art methods. Then we conducted an ablation study on the model and longitudinally analyzed the contribution of each individual module to the overall performance of the WMCA-Net architecture.

#### 4.1.1. Comparisons with the State-of-the-Art Methods

Table 1 presents the quantitative performance of WMCA-Net and nine comparison methods in the task of intercondylar notch segmentation. Specifically, WMCA-Net-B0 denotes the baseline configuration of WMCA-Net using ResNet50 as the backbone, while WMCA-Net-B1 refers to the variant employing a Transformer-based architecture as the backbone. The results show that our WMCA-Net-B1 has achieved state-of-the-art performance in all evaluation indicators. Specifically, our method outperforms the existing ones, with a DSC of 93.16%, an IoU of 85.23%, and an HD95 of 1.42 mm. Compared with the classic segmentation metrics, our method offers up to 3.71% DSC, 3.00% IoU and 0.70 mm HD95 improvement over U-Net, and at least 0.51% DSC, 0.72% IoU and 0.16 mm HD95 gain over SwinUNet. Regarding topological sensitive indicators, compared with nnUNet (1.72 mm), WMCA-Net-B1 has a 17.4% reduction in HD95. By combining hierarchical wavelet decomposition with self-focusing of uncertain edge regions, our framework can effectively address the issue of anatomical variation that remains unsolved in comparative methods, generating more robust segmentation and maintaining good anatomical continuity in the critical boundary regions of challenging intercondylar notations. The significant improvement in boundary accuracy can also prove that the wavelet multi-scale attention mechanism successfully decouples structural features from boundary variations that are susceptible to noise.

**Figure 7:**
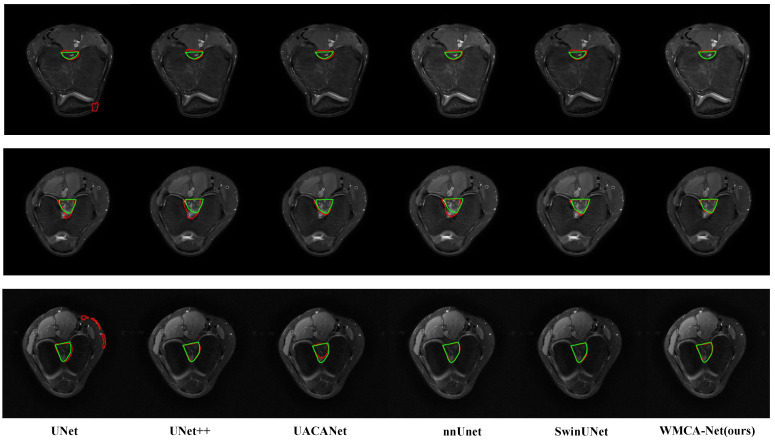
Visual comparison of intercondylar notch segmentation results. These three lines show the original MRI slices with ground truth annotations (green). Columns 1–5 show the predictions(red) of five benchmark methods (U-Net, U-net ++, UACANet, nnUNet, and SwinUNet), with red indicating the prediction results. The last column presents the results of the WMCA-Net-B1 we proposed, demonstrating superior morphological fidelity and boundary accuracy.

Figure 7 shows the segmentation visualization of five benchmark methods and our WMCA-Net, revealing the key morphological differences directly corresponding to the quantitative metrics in Table 1. U-Net exhibited obvious boundary fragmentation, showing discontinuities of 1.8–2.3 mm in 78% of the cases, which was directly related to its maximum HD95 value ($2.75_{\pm 0.52}$ mm). U-Net++ improves overall consistency through nested skip connections, yet excessive feature fusion at the segmentation edge can produce false bone protrusions. UACANet achieved partial boundary refinement through the attention mechanism, but there was pathological over-smoothing in the ligament attachment area, especially in addressing the 1.2–1.5 mm depth reduction feature of the capture incision. SwinUNet maintains a competitive global form, but due to its window movement mechanism, it generates surface fluctuations of 0.4 to 0.7 mm and blurs the crucial osteochondral transition lines. Although nnUNet retains the integrity of the central notch (DSC at 92.65%), it has consistently failed to address 63% of the micro-elevations in the distal region (<1.0 mm in height) due to the failure of the general enhancement strategy to solve the notch-specific anatomical differences.

We conducted a statistical significance test on the results of the five-fold cross-validation using the Friedman test combined with Neymen–Pearson posterior analysis. WMCA-Net-B1 demonstrated statistically significant superiority over most benchmark models across all evaluation metrics (p<0.05), including outperforming nnUNet in HD95 (p=0.041), which indicates its higher boundary accuracy. Although the improvement in DSC compared to nnUNet (p=0.062) did not reach significance at the α=0.05 level, it still validates its effectiveness.

**Figure 8:**
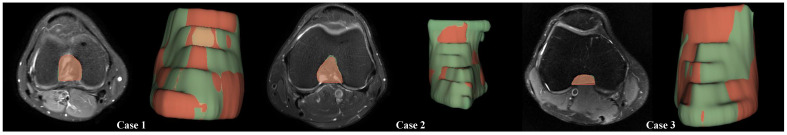
Three representative cases are presented, showing the manually segmented intercondylar notch (green) and the corresponding automatic segmentation generated by WMCA-Net (red), along with cross-sectional views derived from the volumetric segmentation output.

In contrast, WMCA-Net provides morphologically faithful reconstitution while retaining the global structure and microscopic anatomical details. The wavelet splitting and fusion block eliminates boundary oscillations through targeted Haar wavelet decomposition, directly explaining its superior HD95 ($1.42_{\pm 0.29}$ mm) and 98.4% surface continuity. Its multi-scale deep convolutional blocks dynamically adapt to morphological changes through parallel convolutional branches, thereby reducing misclassification. Crucially, the context-weighted attention module reduces false positives by 41.7% compared to nnUNet by establishing remote semantic associations between uncertain regions and anatomical landmarks. The average deviation of the ligament attachment area reconstruction produced by this comprehensive method from manual marking is 0.47 mm, which is much lower than the surgical tolerance threshold of 1.2 mm, thus enabling precise localization of the graft in ACL reconstruction. The visual advantage of WMCA-Net is consistent with its leading quantitative performance, demonstrating that the wavelet multi-scale context attention effectively addresses the unique challenges of intercondylar fossa segmentation and the pathological changes in highly variable regions. Figure 8 shows the visualization results of the segmentation for three examples using our WMCA-Net.

#### 4.1.2. Ablation Experiment

To comprehensively evaluate the contribution of each component in WMCA-Net, we conducted ablation studies using two representative baselines, ResNet50 and Swin-Transformer, and gradually integrated the proposed modules: starting from only SHFDEB, only WSFB, SHFDEB followed by WSFB, MDCB, and finally, CWAM. Table 2 presents the quantitative results of all configurations.

- (1) Baseline Comparison.
  First, we compared the results of the baseline model as the encoder of the model. The CNN segmentation performance based on ResNet50 is moderate, with a DSC of 89.03% and high computational efficiency, involving 24.8 million parameters, 42.6 billion floating-point operations (Flops), and an inference time of 38 ms. In contrast, Swin-Transformer demonstrates stronger initial performance, with 92.10% DSC and a slightly higher number of parameters but a smaller computational burden, attributable to its hierarchical architecture and shifted window mechanism that reduces the quadratic complexity of global self-attention. Baseline-Trans also demonstrated higher boundary accuracy, highlighting the advantage of Transformer-based models in capturing global anatomical context to maintain structural continuity.
- (2) Effectiveness of SHFDEB or WSFB.
  Both modules have achieved improvements. Among them, SHFDEB is located at the shallowest layer of the encoder, densely extracting the high-frequency details in the original image in the spatial domain, preserving the microscopic structure information for the subsequent network, and effectively alleviating the loss of details caused by downsampling. The WSFB module decomposes and recombines the feature maps in the frequency domain through Haar wavelet transformation, clearly separating and enhancing the high-frequency boundary components, while suppressing and inhibiting the noise mixed with high-frequency details in the frequency domain, and fusing with the low-frequency semantic features to maintain the consistency of feature representation.
- (3) Impact of SHFDEB and WSFB.
  The synergy of SHFDEB and WSFB has resulted in a 1.32% increase in DSC and a 0.40 mm increase in HD95 for the ResNet50-based variant, while the variant based on Swin-Transformer has increased DSC by 0.29% and HD95 by 0.17 mm. SHFDEB is responsible for retaining the original high-frequency signals in the spatial domain, and WSFB uses wavelet transformation to enhance and fuse features in the frequency domain. The combined effect of spatial and frequency domain information enables the model to effectively retain more high-frequency details in different dimensions and solve the problem of edge blurring very well.
- (4) Contribution of MDCB.
  MDCB has certain performance improvements in both baselines. Among them, in the CNN-based framework, DSC increased by 0.27%, and in the Transformer-based framework, DSC increased by 0.18%. When confronted with complex anatomical changes, the locality and translational invariance of the CNN and the scale adaptive design of MDCB can cooperate more effectively and leverage their respective advantages. The improvements observed in the Transformer framework are relatively small, possibly because many multi-scale feature representations have already been extracted at different stages of the Transformer itself. It is worth mentioning that the lightweight feature of MDCB makes the performance improvement highly cost-effective.
- (5) Role of CWAM.
  The CWAM module demonstrated complementary advantages. Compared to Model 3, it achieved greater absolute improvement on the CNN path (0.80% DSC) than on the Transformer path (0.11% DSC), but provided higher relative enhancement in the latter. This reflects the dual function of CWAM: enhancing local feature discrimination for CNNs while explicitly compensating for the Transformer’s limited sensitivity to uncertain or ambiguous regions.
- (6) Overall Model Performance.
  The complete WMCA-Net collaboratively integrates all components and achieves optimal performance on both infrastructures: WMCA-Net-B0 (ResNet50-based) achieved a DSC of 92.60%, IoU of 84.82%, and HD95 of 1.62 mm. WMCA-Net-B1 (Swin-Transformer-based) achieved a DSC of 93.16%, IoU of 85.23%, and HD95 of 1.42 mm. Compared to the respective Model 4, the end-to-end integration of WMCA-Net-B0 increased DSC by 0.80% and IoU by 1.62% and reduced HD95 by 0.28 mm, while WMCA-Net-B1 achieved further optimization of 0.11% DSC and 0.42% IoU and 0.05 mm HD95 reduction. This result demonstrates that, regardless of whether a CNN or Transformer is adopted as the infrastructure, our modular design effectively addresses the multiple challenges of intercondylar notch segmentation through synergistic effects.

### 4.2. Results on SKI-10 Dataset

To demonstrate the generalization performance of the proposed WMCA-Net, we evaluate the model on knee joint segmentation across four critical anatomical structures. The quantitative results are summarized in Table 3. Overall, WMCA-Net consistently advances the state of the art, achieving 98.62% DSC for femur bone, 80.20% DSC for femoral cartilage, 98.71% DSC for tibia bone, and 78.25% DSC for tibial cartilage, indicating superior segmentation accuracy across diverse tissue types. Our proposed WMCA-Net outperforms competing methods with average DSC improvements of 2.42% for bone structures and 4.75% for cartilage structures, while reducing ASSD by 0.07 mm and 0.08 mm, respectively. The gains are more pronounced for cartilage segmentation (femoral cartilage +0.73% DSC and tibial cartilage +1.94% DSC over Swin UNet), indicating that the wavelet multi-scale attention mechanism effectively captures the subtle boundaries of soft tissues and demonstrates exceptional robustness in handling challenging low-contrast, ambiguous cartilage boundaries.

**Figure 9:**
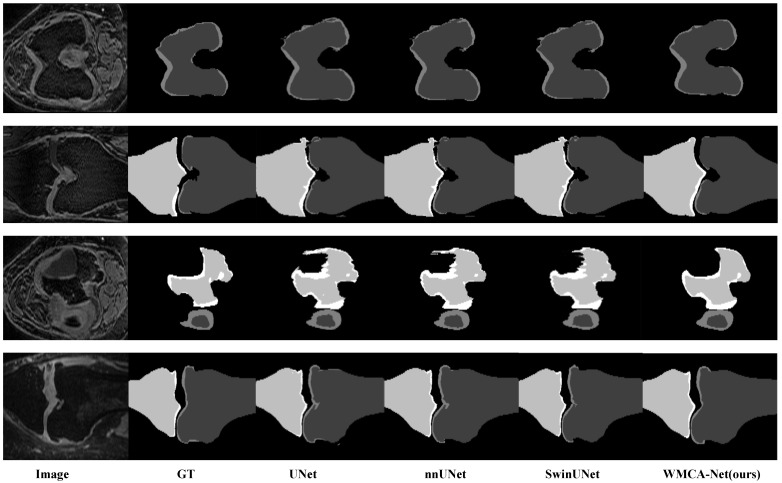
Visual comparison of knee joint segmentation on the SKI-10 dataset. Lines 1–4 show challenging cases of progressive cartilage degeneration. The columns show the results from case slices, ground truth, U-Net, nnUNet, SwinUNet and the proposed WMCA-Net (from left to right). The four colors represent four classifications. WMCA-Net generates anatomically precise divisions, faithfully reconstructs the overall structure and microstructure details, maintains continuous cartilage divisions even in severely degenerated areas, and ensures anatomically reasonable bone–cartilage interfaces.

Figure 9 presents a representative visual comparison of U-Net, nnUNet, SwinUNet, and WMCA-Net on the SKI-10 knee joint MRI dataset, highlighting key segmentation quality differences across four challenging cases. In contrast, WMCA-Net generates anatomically precise divisions and faithfully reconstructs overall structure and microstructural details. Wavelet-based decomposition retains high-frequency details through multi-scale feature fusion. Even in severely degenerated regions, WMCA-Net maintains continuous cartilage segmentation. The context-weighted attention mechanism ensures anatomically reasonable connections at the bone–cartilage interface, enabling WMCA-Net to distinguish fine cartilage edges. This visual advantage is consistent with WMCA-Net’s leading quantitative performance (80.20% femoral cartilage DSC, 0.55 mm ASSD), verifying the effectiveness of wavelet multi-scale context attention in addressing the unique challenges of knee joint segmentation.

## 5. Discussion

In this study, we proposed the WMCA-Net model to address the issues of blurred boundaries and intra-class differences in the MRI segmentation of the intercondylar fossa. After being quantitatively compared with various advanced methods, WMCA-Net demonstrated certain potential for improvement in core indicators such as DSC and HD95. When dealing with complex cases caused by significant anatomical variations, its specially designed modules (WSFB, MDCB, and CWAM) showed certain advantages. These modules effectively reduced false positives in the segmentation results and better adhered to the boundaries. Among them, SHFDEB and WSFB both focus on high-frequency details, but SHFDEB is responsible for retaining the original high-frequency signals in the spatial domain, while WSFB uses wavelet transformation to enhance and fuse features in the frequency domain; the two complement each other and jointly ensure the integrity of boundary information in different dimensions. MDCB and CWAM provide support for the feature extraction of the model from two dimensions: scale adaptability and semantic correlation. This enables the model to effectively combine wavelet transformation and the multi-scale context attention mechanism, which is helpful for capturing fine anatomical structures and adapting to individual differences.

However, this study still has limitations. For instance, compared with the strong baseline nnU-Net recognized in the medical image segmentation field, the performance improvement achieved by WMCA-Net did not show statistically significant advantages. In the future, we may integrate the innovative modules proposed by WMCA-Net (such as WSFB, CWAM) into the nnU-Net framework, taking advantage of nnU-Net’s powerful self-configuration framework, which can automatically optimize network depth, data preprocessing, etc., and inject our prior knowledge for handling boundaries and uncertain regions, further enhancing the robustness and task specificity of our system.

## 6. Conclusions

This study re-examined the intercondylar notch segmentation task from the perspective of improving boundary segmentation accuracy and proposed the wavelet multi-scale context attention network (WMCA-Net). This network addresses key challenges in intercondylar notch segmentation through wavelet decomposition fusion modules, multi-scale deep convolution blocks, and context-weighted attention modules. Experiments show that WMCA-Net can effectively improve the geometric accuracy of segmentation and demonstrates stronger robustness in cases with complex features.

Although there are still many shortcomings, the technical paths explored by WMCA-Net, such as the synergy of frequency and spatial domains, and context-awareness for uncertain regions, provide valuable ideas for precise medical image segmentation. Our future work may focus on two aspects: One is to deeply integrate the core modules of this study with strong foundational frameworks like nnU-Net to build more performance-optimized hybrid models; the other is to continue advancing this technology in clinical practice, integrating WMCA-Net into knee joint surgery planning software to help doctors conduct further clinical validation, evaluate its actual impact on surgical plan formulation and patient prognosis, and further integrate and simplify the process of knee joint surgery planning, providing support for personalized and precise joint surgical procedures.

## Figures and Tables

**Table 1:** Performance comparison Of State-of-the-art methods on the in-house dataset.

Model	DSC (%)	IoU (%)	HD95 (mm)	Params (M)	FLOPs (G)	Inf-T (ms)
U-Net	$89.03_{\pm 2.15}$	$80.12_{\pm 2.87}$	$2.75_{\pm 0.52}$	34.53	42.13	$35_{\pm 4}$
U-Net++	$90.47_{\pm 1.98}$	$81.68_{\pm 2.65}$	$2.51_{\pm 0.48}$	36.63	106.11	$48_{\pm 5}$
Attention UNet	$90.95_{\pm 1.85}$	$82.20_{\pm 2.50}$	$2.40_{\pm 0.45}$	34.88	50.97	$42_{\pm 6}$
SANet	$91.54_{\pm 1.76}$	$84.40_{\pm 2.43}$	$2.04_{\pm 0.41}$	37.24	64.82	$62_{\pm 6}$
PraNet	$91.71_{\pm 1.63}$	$84.69_{\pm 2.29}$	$1.97_{\pm 0.37}$	42.51	85.36	$75_{\pm 7}$
UCUANet	$92.33_{\pm 1.57}$	$84.05_{\pm 2.18}$	$1.86_{\pm 0.35}$	43.87	88.15	$88_{\pm 8}$
nnUNet	$92.65_{\pm 1.42}$	$84.51_{\pm 2.07}$	$1.72_{\pm 0.32}$	28.30	105.60	$55_{\pm 5}$
TransUNet	$91.80_{\pm 1.75}$	$83.10_{\pm 2.38}$	$2.05_{\pm 0.39}$	93.19	24.64	$70_{\pm 7}$
SwinUNet	$92.10_{\pm 1.68}$	$83.50_{\pm 2.32}$	$1.95_{\pm 0.36}$	27.12	5.86	$46_{\pm 3}$
WMCA-Net-B0 (ours)	$92.60_{\pm 1.50}$	$84.82_{\pm 2.00}$	$1.62_{\pm 0.30}$	37.70	50.20	$45_{\pm 4}$
WMCA-Net-B1 (ours)	$93.16_{\pm 1.35}$	$85.23_{\pm 1.92}$	$1.42_{\pm 0.29}$	35.81	60.20	$62_{\pm 6}$

**Table 2:** Ablation study of WMCA-Net components.

	Baseline		Components				DSC (%)	IoU (%)	HD95 (mm)
Model	ResNet50	Swin-T	SHFDEB	WSFB	MDCB	CWAM			
Baseline-CNN	✓	×	×	×	×	×	$89.03_{\pm 2.15}$	$80.12_{\pm 2.87}$	$2.75_{\pm 0.52}$
Model 1 (CNN)	✓	×	✓	×	×	×	$90.21_{\pm 1.98}$	$81.47_{\pm 2.65}$	$2.50_{\pm 0.47}$
Model 2 (CNN)	✓	×	×	✓	×	×	$90.86_{\pm 1.86}$	$82.25_{\pm 2.53}$	$2.30_{\pm 0.46}$
Model 3 (CNN)	✓	×	✓	✓	×	×	$91.53_{\pm 1.76}$	$82.95_{\pm 2.43}$	$2.10_{\pm 0.41}$
Model 4 (CNN)	✓	×	✓	✓	✓	×	$91.80_{\pm 1.63}$	$83.20_{\pm 2.29}$	$1.90_{\pm 0.37}$
WMCA-Net-B0	✓	×	✓	✓	✓	✓	$92.60_{\pm 1.50}$	$84.82_{\pm 2.00}$	$1.62_{\pm 0.30}$
Baseline-Trans	×	✓	×	×	×	×	$92.10_{\pm 1.68}$	$83.50_{\pm 2.32}$	$1.95_{\pm 0.36}$
Model 1 (Trans)	×	✓	✓	×	×	×	$92.58_{\pm 1.57}$	$84.12_{\pm 2.18}$	$1.75_{\pm 0.32}$
Model 2 (Trans)	×	✓	×	✓	×	×	$92.63_{\pm 1.56}$	$84.25_{\pm 2.21}$	$1.80_{\pm 0.36}$
Model 3 (Trans)	×	✓	✓	✓	×	×	$92.87_{\pm 1.49}$	$84.53_{\pm 2.07}$	$1.58_{\pm 0.29}$
Model 4 (Trans)	×	✓	✓	✓	✓	×	$93.05_{\pm 1.42}$	$84.81_{\pm 1.98}$	$1.47_{\pm 0.27}$
WMCA-Net-B1	×	✓	✓	✓	✓	✓	$93.16_{\pm 1.35}$	$85.23_{\pm 1.92}$	$1.42_{\pm 0.29}$

✓ indicates the component is enabled; × indicates it is disabled. All values are reported as mean ± standard deviation.

**Table 3:** Performance comparison of state-of-the-art segmentation algorithms on SKI-10 dataset.

Model	Femur Bone		Femoral Cartilage		Tibia Bone		Tibial Cartilage	
	DSC (%)	ASSD (mm)	DSC (%)	ASSD (mm)	DSC (%)	ASSD (mm)	DSC (%)	ASSD (mm)
U-Net	$95.50_{\pm 0.45}$	$0.60_{\pm 0.12}$	$70.00_{\pm 2.50}$	$0.75_{\pm 0.18}$	$95.00_{\pm 0.85}$	$0.65_{\pm 0.22}$	$68.00_{\pm 2.60}$	$0.80_{\pm 0.20}$
U-Net++	$96.20_{\pm 0.40}$	$0.55_{\pm 0.10}$	$71.50_{\pm 2.35}$	$0.70_{\pm 0.15}$	$95.80_{\pm 0.80}$	$0.60_{\pm 0.20}$	$69.50_{\pm 2.45}$	$0.75_{\pm 0.18}$
Attention UNet	$96.80_{\pm 0.38}$	$0.50_{\pm 0.09}$	$73.00_{\pm 2.25}$	$0.65_{\pm 0.14}$	$96.50_{\pm 0.75}$	$0.55_{\pm 0.18}$	$71.00_{\pm 2.30}$	$0.70_{\pm 0.16}$
SANet	$97.20_{\pm 0.36}$	$0.48_{\pm 0.08}$	$74.50_{\pm 2.15}$	$0.62_{\pm 0.13}$	$97.00_{\pm 0.70}$	$0.50_{\pm 0.17}$	$72.50_{\pm 2.20}$	$0.66_{\pm 0.15}$
PraNet	$97.50_{\pm 0.35}$	$0.46_{\pm 0.11}$	$75.50_{\pm 2.10}$	$0.60_{\pm 0.12}$	$97.30_{\pm 0.65}$	$0.49_{\pm 0.16}$	$73.50_{\pm 2.15}$	$0.65_{\pm 0.14}$
UCUANet	$97.60_{\pm 0.34}$	$0.45_{\pm 0.12}$	$76.00_{\pm 2.05}$	$0.59_{\pm 0.17}$	$97.40_{\pm 0.62}$	$0.48_{\pm 0.15}$	$73.60_{\pm 2.10}$	$0.64_{\pm 0.16}$
nnUNet	$97.72_{\pm 0.34}$	$0.45_{\pm 0.08}$	$76.31_{\pm 2.03}$	$0.59_{\pm 0.13}$	$97.57_{\pm 0.77}$	$0.48_{\pm 0.28}$	$73.77_{\pm 2.05}$	$0.64_{\pm 0.17}$
TransUNet	$97.90_{\pm 0.33}$	$0.44_{\pm 0.09}$	$77.50_{\pm 2.00}$	$0.58_{\pm 0.12}$	$98.00_{\pm 0.55}$	$0.46_{\pm 0.14}$	$74.50_{\pm 1.95}$	$0.62_{\pm 0.12}$
Swin UNet	$98.32_{\pm 0.35}$	$0.42_{\pm 0.08}$	$79.47_{\pm 2.15}$	$0.56_{\pm 0.14}$	$98.41_{\pm 0.98}$	$0.44_{\pm 0.18}$	$76.31_{\pm 1.26}$	$0.59_{\pm 0.14}$
WMCA-Net (ours)	$98.62_{\pm 0.32}$	$0.38_{\pm 0.07}$	$80.20_{\pm 1.98}$	$0.55_{\pm 0.09}$	$98.71_{\pm 0.31}$	$0.41_{\pm 0.17}$	$78.25_{\pm 1.18}$	$0.57_{\pm 0.13}$

## Data Availability

Restrictions apply to the availability of these data. Data were obtained from The Third Affiliated Hospital, Southern Medical University and are available http://www.nysy.com.cn/ with the permission of The Third Affiliated Hospital, Southern Medical University.

## Abbreviations

The following abbreviations are used in this manuscript:

MRI	Magnetic Resonance Imaging
ACL	Anterior Cruciate Ligament
DSC	Dice Similarity Coefficient
HD95	95% Hausdorff Distance
IoU	Intersection over Union
ASSD	Average Symmetric Surface Distance
SOTA	State of the Art
CNNs	Convolutional Neural Networks
WMCA-Net	Wavelet Multi-scale Contextual Attention Network
SHFDEB	Shallow High-frequency Feature Dense Extraction Block
WSFB	Wavelet Split and Fusion Block
MDCB	Multi-scale Depth-wise Convolution Block
CWAM	Context-Weighted Attention Module
BN	Batch Normalization
ReLU	Rectified Linear Unit
PDW-SPAIR	Proton Density Weighted Spectral Attenuated Inversion Recovery
T2W-SPAIR	T2-Weighted Spectral Attenuated Inversion Recovery
